# Supplementary material for: Identification of photoperiod-induced specific miRNAs in the adrenal glands of Sunite sheep (Ovis aries)
Source: Front Vet Sci. 2022 Jul 22;9:888207. doi: 10.3389/fvets.2022.888207 (PMC9354845; doi:10.3389/fvets.2022.888207)
Supplement: Supplementary file 1 [file Data_Sheet_1.ZIP › Supplementary file/miRDeep2 file.zip!/miRDeep2 file/Novel_Structure/LP42A3/Novel_484.pdf]

Provisional ID	:	NC_019472.2_16581
Score total	:	2
Score for star read(s)	:	-1.3
Score for read counts	:	0
Score for mfe	:	1.7
Score for randfold	:	1.6
Score for cons. seed	:	
Total read count	:	157
Mature read count	:	157
Loop read count	:	0
Star read count	:	0

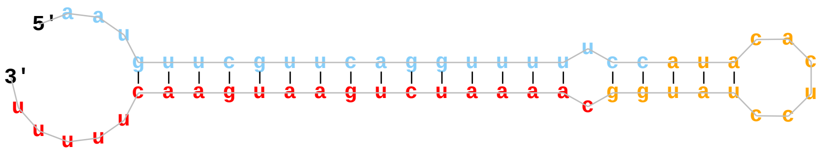

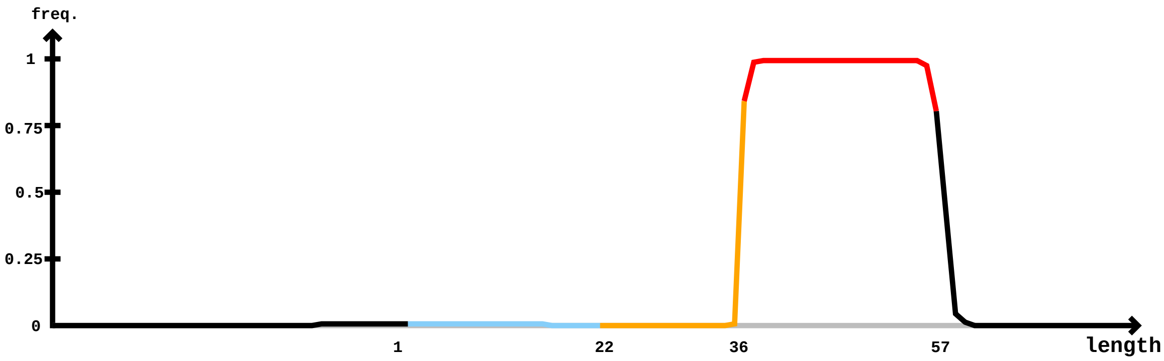


```
                                                   Star                                 Mature
5'- ugaagcacaaaaagcuuauguauuggguuggccaaaauguucguucagguuuuuccauacacuccuauggcaaaaucugaaugaacuuuuugcccauuugaacucaaucac   -3'    exp
    ..((((.......)))).((.((((((((((.(((((.(((((((((((((((.(((((......))))).))))))))))))))).))))).))).....)))))))))).    reads      mm       sample
    ..........................guuggccaaaauguucguucaggu.............................................................    1          0        seq
    .....................................................................gAaaaaucugaaugaacuuuuu....................    1          1        seq
    ......................................................................Aaaaaucugaaugaacuuuu.....................    23         1        seq
    ......................................................................Aaaaaucugaaugaacuuuuu....................    65         1        seq
    ......................................................................caaaaucugaaugaacuuuuu....................    2          0        seq
    ......................................................................Aaaaaucugaaugaacuuuuug...................    42         1        seq
    .......................................................................aaaaucugaaugaacuuu......................    3          0        seq
    .......................................................................aaaaucugaaCgaacuuuu.....................    3          1        seq
    .......................................................................aaaaucCgaaugaacuuuu.....................    1          1        seq
    .......................................................................aaaaucugaaugaacuuuuA....................    1          1        seq
    .......................................................................aaaaucugaaugaGcuuuuu....................    2          1        seq
    .......................................................................aaaaucCgaaugaacuuuuug...................    1          1        seq
    .......................................................................aaaaucugaaugaacuuuuuU...................    5          1        seq
    .......................................................................aaaaucAgaaugaacuuuuug...................    1          1        seq
    .......................................................................aaaaucugaaugaacuuuuGgc..................    1          1        seq
    .......................................................................aaaaucugaaugaacuuuuugc..................    1          0        seq
    .......................................................................aaaaCcugaaugaacuuuuugc..................    2          1        seq
    .......................................................................aaaaucugaaugaacuuuuugGc.................    2          1        seq
    ........................................................................aaaucugaaugaacuuuuGgc..................    1          1        seq
```
